# Effects of Nonpharmacological and Nonsurgical Intervention for Lower Urinary Tract Symptoms in Parkinson’s Disease: A Systematic Review and Meta-analysis

**DOI:** 10.1016/j.euros.2026.03.009

**Published:** 2026-04-09

**Authors:** Hiroyuki Ohtsuka, Mifuka Ouchi, Tomoko Otsuka, Takeya Kitta, Daisuke Yoneoka, Ryuji Sakakibara

**Affiliations:** a Graduate School of Health Sciences, Showa University, Kanagawa, Japan; b Department of Rehabilitation, School of Nursing and Rehabilitation Sciences, Showa University, Kanagawa, Japan; c Institute of Clinical Epidemiology, Showa University, Kanagawa, Japan; d Department of Renal and Genitourinary Surgery, Graduate School of Medicine, Hokkaido University, Hokkaido, Japan; e Department of Nursing, Faculty of Healthcare Sciences, Chiba Prefectural University of Health Sciences, Chiba, Japan; f Department of Renal and Urologic Surgery, Asahikawa Medical University, Hokkaido, Japan; g Center for Surveillance, Immunization, and Epidemiologic Research, National Institute of Infectious Diseases, Tokyo, Japan; h Neurology Clinic Tsudanuma and Dowakai Chiba Hospital, Chiba, Japan

**Keywords:** Lower urinary tract symptoms, Meta-analysis, Non-pharmacological and non-surgical intervention, Parkinson’s disease, Systematic review

## Abstract

**Background and objective:**

Lower urinary tract symptoms (LUTS) are common in Parkinson’s disease (PD) and reduce quality of life. We assessed whether nonpharmacological and nonsurgical (NPNS) interventions improve PD-related LUTS.

**Methods:**

We searched PubMed, CENTRAL, PEDro, and CINAHL from inception to November 28, 2025, and screened reference lists. Randomized controlled trials (RCTs) and nonrandomized studies in adults with PD comparing NPNS interventions with no intervention, sham/placebo, or usual care were eligible. Primary outcomes were 24-h bladder-diary episodes (voids, urgency, incontinence). Secondary outcomes were validated symptom, bother, and urinary-related quality-of-life (QOL) scales. Random-effects meta-analyses used postintervention values. Risk of bias (RoB) was assessed with Cochrane RoB 2/RoB Assessment Tool for Non-randomized Studies, and certainty with the Grading of Recommendations, Assessment, Development and Evaluations approach.

**Key findings and limitations:**

*S*ixteen studies were included (13 RCTs, two nonrandomized studies, one case study). Urinary incontinence episodes decreased modestly (mean difference −0.91 episodes/24 h, 95% confidence interval −1.74 to −0.08; moderate certainty). For urgency episodes, symptom scores, and bother scores, estimates suggested possible benefit but were imprecise (low certainty). Effects on voiding frequency, urinary-related QOL, and the non-motor symptoms scale urinary domain were highly uncertain (very low certainty). Many outcomes showed substantial heterogeneity; pooled estimates should be interpreted as conditional summaries. Publication bias could not be assessed because most meta-analyses included fewer than 10 studies.

**Conclusions and clinical implications:**

NPNS interventions may improve some LUTS outcomes in PD, with the most consistent signal for urinary incontinence. More adequately powered trials enrolling patients with confirmed LUTS and using standardized LUTS-specific outcomes, follow-up, and comparator definitions are needed.

## Take Home Message

Non-pharmacological and non-surgical interventions may provide benefit for selected lower urinary tract symptoms in people with Parkinson’s disease, with the most consistent signal observed for urinary incontinence. However, confidence in effects varies substantially across outcomes and is limited by imprecision and between-study heterogeneity. These interventions may be considered as low-risk, patient-centered options alongside standard drug treatments, but pooled estimates should be interpreted cautiously as conditional summaries rather than definitive effects.

ADVANCING PRACTICE **What does this study add?**

This study provides a comprehensive synthesis of non-pharmacological and non-surgical interventions for lower urinary tract symptoms in Parkinson’s disease. It identifies a modest and more consistent signal for urinary incontinence, while effects on other symptom domains, bother, and quality of life were variable and often imprecise. By integrating heterogeneity and certainty-of-evidence assessments, this review clarifies that pooled estimates should be interpreted as conditional summaries rather than definitive treatment effects, thereby informing cautious, outcome-specific clinical decision-making and future trial design.

**Clinical Relevance**

Nonpharmacological and nonsurgical interventions may be considered as part of a comprehensive management strategy for lower urinary tract symptoms in Parkinson’s disease, particularly for urinary incontinence and in patients for whom reducing medication burden is important. These findings should be interpreted cautiously, as the certainty of evidence was low to very low for most outcomes and further adequately powered trials using standardized LUTS-specific outcomes are needed. Associate Editor: Véronique Phé.

**Patient Summary**

This study reviewed non-drug and non-surgical treatments for bladder symptoms in people with Parkinson’s disease. Some approaches, such as behavioral therapies, may help reduce certain bladder symptoms, particularly urinary incontinence, but the size and consistency of benefits varied across studies. Other treatments, including electrical stimulation of a nerve near the ankle, showed uncertain effects on symptom frequency but may influence how bothersome symptoms feel for some people. Overall, these treatments may be considered as additional options alongside standard care, although more research is needed to better understand who benefits most.

## 1 Introduction

Parkinson’s disease (PD) is a progressive neurodegenerative disorder with both motor and nonmotor symptoms that impact quality of life (QOL) [1], [2]. Among nonmotor symptoms, autonomic dysfunctions like lower urinary tract symptoms (LUTS) and constipation are particularly burdensome from the early stages [3], [4]. LUTS are present in 37–71% of patients and commonly include urgency, frequency, and incontinence [2], [5], [6]. These symptoms negatively impact sleep and daytime activities, contributing to a reduction in QOL, an increased risk of falls, caregiver distress, and early institutionalization [7], [8], [9], [10], [11], highlighting the need for effective interventions.

Anticholinergics are often prescribed but have side effects such as constipation, dry mouth, falls, delirium, and cognitive decline [12], [13], [14], [15]. While selective beta-agonists have been introduced, polypharmacy remains a concern [16], [17]. Therefore, interest has grown in nonpharmacological and nonsurgical (NPNS) interventions, which include lifestyle interventions, bladder training, and physiotherapy, and carry low side-effect risks and may improve QOL [18], [19], [20]. Strategies such as noninvasive brain or peripheral nerve stimulation modulate neural activity to enhance urinary function with fewer adverse effects than drugs [21], [22], [23], [24]. Despite promising outcomes, few studies have targeted PD-specific LUTS. This systematic review and meta-analysis aimed to evaluate the effectiveness of NPNS interventions for LUTS in PD, particularly regarding QOL.

## 2 Methods

### 2.1 Data sources and search strategy

Following the Preferred Reporting Items for Systematic Reviews and Meta-analyses (PRISMA) statement, this systematic review was registered with the International Prospective Register of Systematic Reviews (PROSPERO) database on September 9, 2021 (registration number: CRD42021269383) [25]. We conducted and reported this review in accordance with PRISMA 2020 and the European Urology guidance for systematic reviews; given the limited number of observational studies, we did not additionally apply Meta-analysis Of Observational Studies in Epidemiology (MOOSE) guidelines. Several databases, including PubMed, Cochrane Central Register of Controlled Trials (CENTRAL), CINAHL, and PEDro, were searched from their inception to November 28, 2025. The search was initially conducted in September 2021 and subsequently updated in September 2024 and November 2025 to ensure currency; however, the present report reflects the final updated search (November 28, 2025). The search included studies involving patients of all ages, genders, and ethnicities, focusing on key terms related to the target population, interventions, and outcomes, combined using ‘AND’ and ‘OR’. The bibliographies of relevant studies, reviews, and other meta-analyses were manually searched for additional studies. Details of the search strategy and update history are provided in Supplementary Appendix S1–S2.

A table listing full-text articles excluded after eligibility assessment, together with reasons for exclusion, is provided in the Supplementary Appendix S3.

### 2.2 Inclusion and exclusion criteria

The selection criteria included randomized controlled trials (RCTs), non-RCTs, cohort studies, case–control studies, cross-sectional studies, case reports, and case series. Eligible participants were patients with PD or dementia with Lewy bodies (DLB). The language of the studies was limited to English. Studies were excluded if they were review articles, conference abstracts, or letters to the editor or involved individuals with Parkinsonism other than PD or DLB.

### 2.3 Comparators (control conditions)

We categorized control conditions as (1) no intervention/wait-list, (2) sham stimulation/placebo, (3) usual care/standard care (including continuation of stable medication), and (4) other active comparators where applicable (eg, pharmacological-only comparator). Comparator category was extracted for each study and considered a potential source of clinical/methodological heterogeneity in interpretation of pooled effects.

### 2.4 Screening and data extraction

Two independent reviewers (HO, MO, TO, TT, and RS) screened studies for eligibility and conducted full-text reviews. For data extraction, two independent reviewers (HO and MO) used a preformulated data collection form to systematically gather information on study characteristics, participant details, interventions, outcomes, and other relevant factors.

### 2.5 Outcomes

The primary outcomes were bladder-diary event counts standardized to a 24-hr metric and pooled only when the underlying construct was equivalent across studies. We defined (1) voiding frequency as the number of micturitions per 24 hr, (2) urgency episodes as the number of urgency events per 24 hr, and (3) urinary incontinence episodes as the number of leakage events per 24 hr. In this manuscript, we use the term “episodes” only for urgency and incontinence; voiding frequency is reported as “voids per 24 hr.” When diary outcomes were reported as totals over multiple days, we converted them to per-24-hr values using predefined rules, with standard deviations (SDs) converted accordingly. When an outcome was derived (eg, by summing daytime and nocturnal voids), it was retained as “total daily voids” and flagged for sensitivity analysis.

Secondary outcomes were symptom, bother, and disease-specific urinary QOL measured by validated questionnaires. For all outcomes, we coded the direction of benefit so that negative effect estimates indicated improvement with the intervention. If a scale was oriented such that higher scores indicated better status, values were multiplied by −1 prior to pooling. We applied predefined operational rules for all outcomes, including scale orientation, direction-of-benefit coding, and unit harmonization. To avoid multiplicity, a hierarchy for selecting one measure per outcome domain was established.

Full instrument-level outcome specifications are available in Supplementary Appendix S4.

### 2.6 Risk of bias and quality assessment

The risk of bias (RoB) in included studies was assessed using version 2 of the Cochrane RoB tool for randomized trials (RoB 2) and the RoB Assessment tool for Non-randomized Studies (RoBANS) [26], [27]. The quality and certainty of evidence were evaluated using the Grading of Recommendations, Assessment, Development and Evaluations (GRADE) approach [28].

### 2.7 Protocol and deviations

This review was prospectively registered in PROSPERO, and the conduct broadly followed the registered methods. In accordance with PRISMA item 24c, deviations from the registered protocol were explicitly documented and justified. Key deviations included refinements of operational outcome definitions, feasibility-related limitations of prespecified subgroup and sensitivity analyses, incorporation of GRADE, clarification of publication intent, and updated literature searches.

Detailed protocol deviations are summarized in Supplementary Appendix S5.

### 2.8 Statistical analyses

Meta-analyses were performed in RevMan 5.4 using a random-effects model because clinical and methodological heterogeneity was expected. For continuous outcomes, mean difference (MD) was used when outcomes were reported on the same scale and unit, and standardized mean difference (SMD) was used only when conceptually similar outcomes were measured using different instruments. Primary analyses were based on postintervention values; change scores were used only when postintervention data were unavailable. Prespecified sensitivity analyses were conducted where feasible, including analyses related to derived diary outcomes and imputed SDs. Statistical heterogeneity was assessed using I^2^ and τ^2^ and interpreted primarily based on confidence intervals (CIs) rather than thresholds alone.

Additional sensitivity analyses are described in Supplementary Appendix S6.

## 3 Results

### 3.1 Study selection

**Fig. 1:**
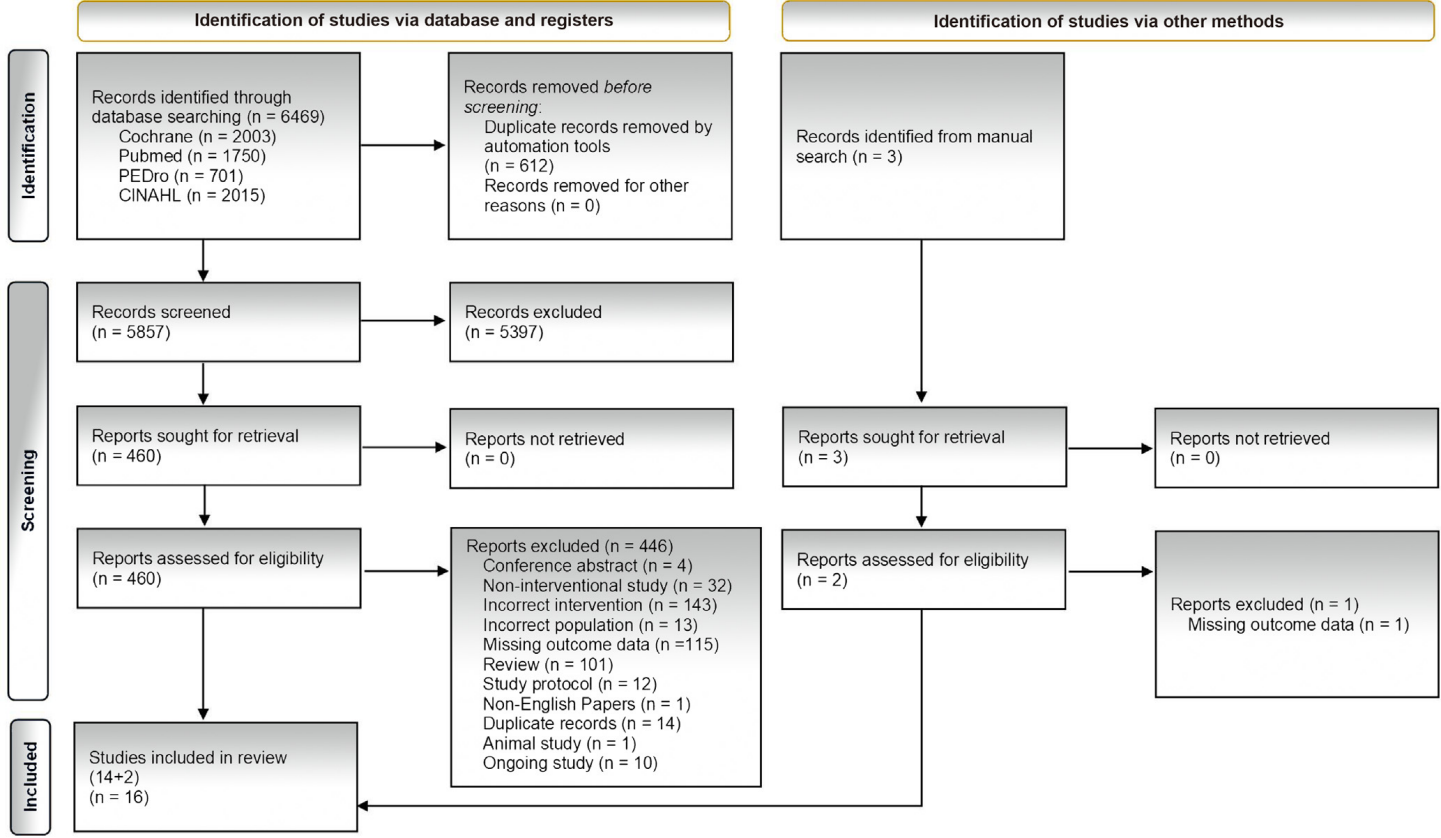
Flowchart of search strategy, presented as a PRISMA diagram.

A total of 6469 records were identified through database searches. After screening titles, abstracts, and full texts, 16 studies met the inclusion criteria (Fig. 1). No studies focused specifically on DLB. Reasons for full-text exclusion are provided in Supplementary Appendix S3.

### 3.2 Study characteristics

Sixteen reports were included, comprising 13 RCTs, two nonrandomized pre–post studies, and one case study. Across the RCTs, 903 participants were enrolled; the two nonrandomized studies included 25 participants, and the case study included one participant. Interventions included lifestyle interventions [29], [30], [31], [32], behavioral therapy [33], [34], [35], [36], transcutaneous tibial nerve stimulation (TTNS) [37], [38], [39], [40], [41], noninvasive brain stimulation [42], caloric vestibular stimulation [43], and a multidisciplinary telemedicine-based rehabilitation program [44]. Outcomes included bladder-diary measures, validated urinary symptoms, and bother questionnaires, urinary-related QOL instruments, and nonmotor symptom scales with urinary domains. Detailed study characteristics, comparator conditions, outcome definitions, and adverse event reporting are summarized in Supplementary Appendix S8.

### 3.3 RoB

**Fig. 2:**
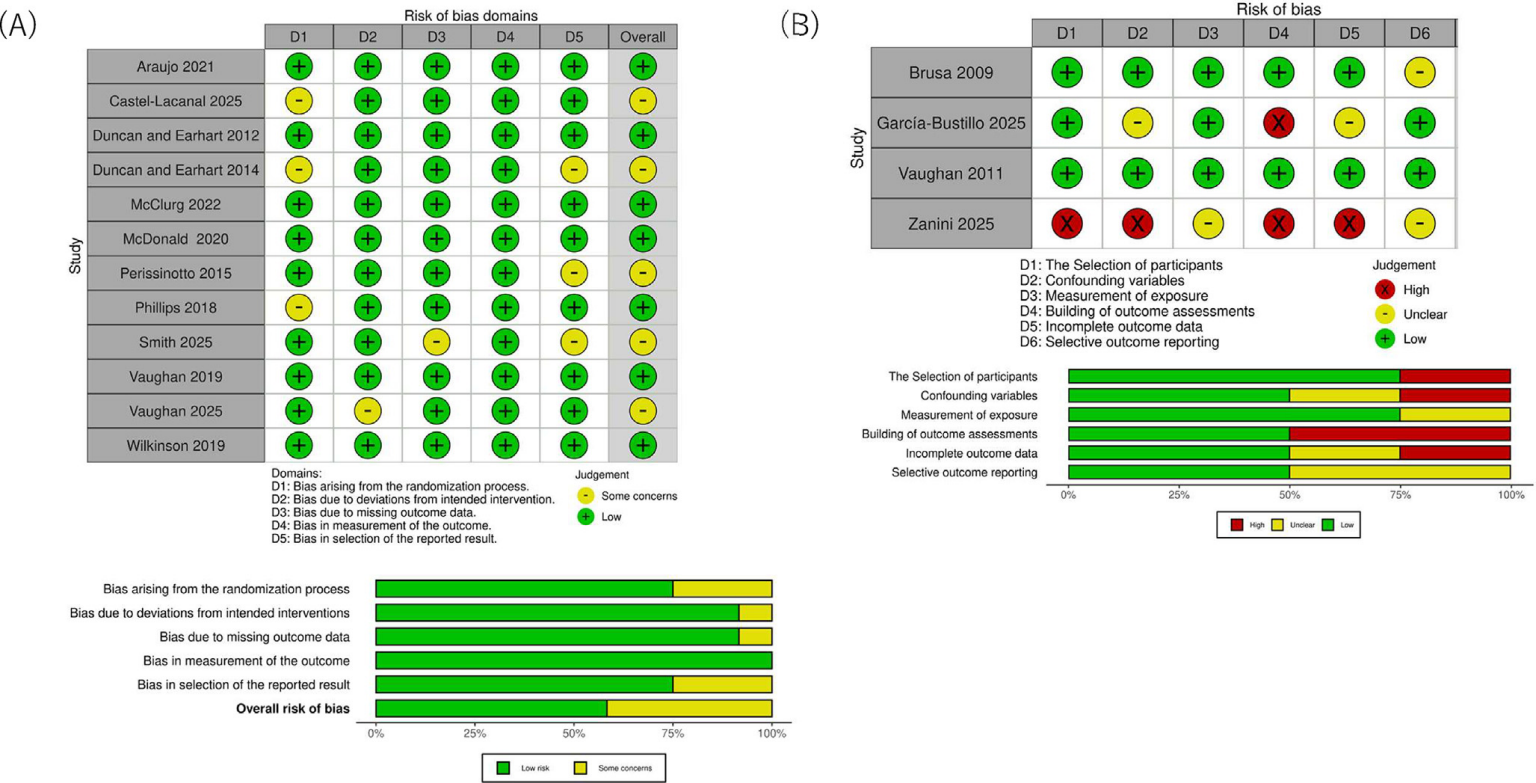
Risk of bias is presented as percentages across all included studies. (A) The Cochrane Risk of Bias 2 (RoB 2) tool was used to assess the risk of bias. Green indicates low risk of bias, red indicates high risk of bias, and yellow indicates some concerns about risk of bias. (B) The Risk of Bias Assessment Tool for Non-randomized Studies (RoBANS) was used to assess the risk of bias. Green indicates low risk of bias and yellow indicates unclear risk of bias.

RoB in RCTs was assessed using the RoB 2 tool (Fig. 2A). Seven RCTs were judged to be at low RoB, and five were judged to have some concerns, mainly related to the randomization process and selection of the reported results. Four nonrandomized studies were evaluated using RoBANS (Fig. 2B).

### 3.4 Integration of results

Meta-analyses were performed for outcomes reported by 10 RCTs with available data [29], [30], [31], [33], [35], [37], [38], [39], [41], [43]. Two RCTs were excluded from meta-analysis because outcome data were unavailable or because a noninferiority design with an active pharmacological comparator was not compatible with superiority-based effect estimation [36], [40]. Statistical heterogeneity ranged from low to considerable across pooled outcomes (I^2^ = 18–81%). Absolute between-study variance (τ^2^) was therefore reported alongside I^2^ to support clinically interpretable assessment of heterogeneity. Because control conditions differed across studies, pooled estimates were interpreted with attention to comparator heterogeneity as a potential contributor to between-study variability. Exploratory subgroup analyses by intervention type, comparator category, assessment timing, and RoB category were conducted to support interpretation of between-study heterogeneity and are reported in the Supplementary Appendix S9.

### 3.5 Primary outcomes

#### 3.5.1 Number of voids per 24 hr

**Fig. 3:**
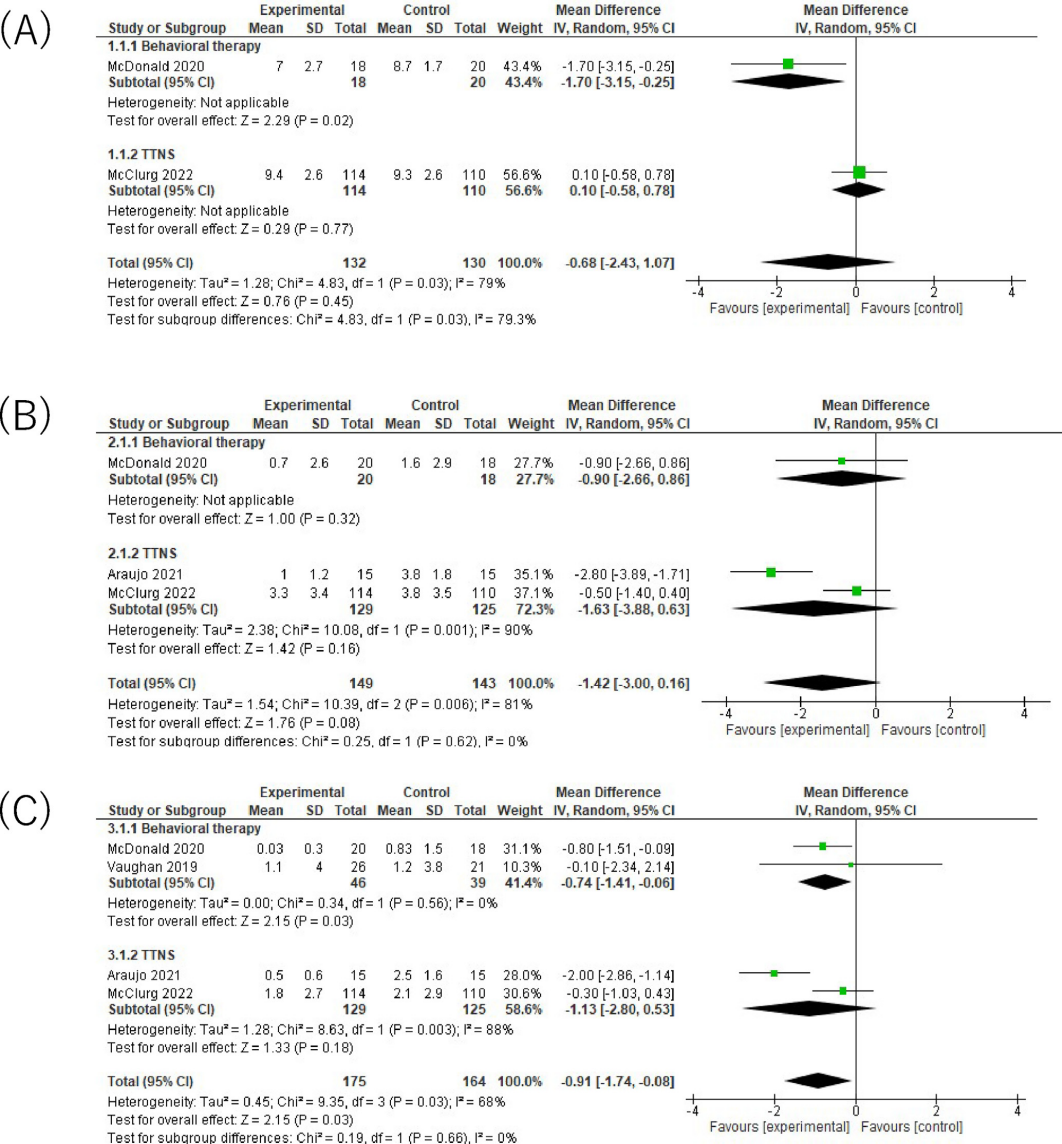
Forest plots analysis on the outcome of bladder diaries. (A) Voids per 24 hr (voiding frequency). (B) Urgency episodes per 24 hr. (C) Urinary incontinence episodes per 24 hr.

For voids per 24 hr, the pooled estimate favored NPNS interventions, although the CI was wide and included no important difference (MD −0.68, 95% CI −2.43 to 1.07; Fig. 3A) [33], [39]. Between-study heterogeneity was considerable (τ^2^ = 1.28, I^2^ = 79%), indicating substantial absolute variability in effect sizes across trials. The certainty of evidence for this outcome was very low.

#### 3.5.2 Episodes of urgency

For urgency episodes per 24 hr, the pooled estimate was imprecise, with the 95% CI spanning both benefit and no important difference (MD −1.42, 95% CI −3.00 to 0.16; Fig. 3B) [33], [37], [39]. Heterogeneity was considerable (τ^2^ = 1.54, I^2^ = 81%), reflecting large between-study variability. The certainty of evidence for urgency episodes was low.

#### 3.5.3 Episodes of incontinence

For urinary incontinence episodes per 24 hr, the pooled estimate suggested a modest reduction with NPNS interventions (MD −0.91, 95% CI −1.74 to −0.08; Fig. 3C) [33], [35], [37], [39]. Heterogeneity was moderate (τ^2^ = 0.45, I^2^ = 68%). The certainty of evidence for incontinence episodes was moderate.

### 3.6 Secondary outcomes

#### 3.6.1 Urinary symptom scores

**Fig. 4:**
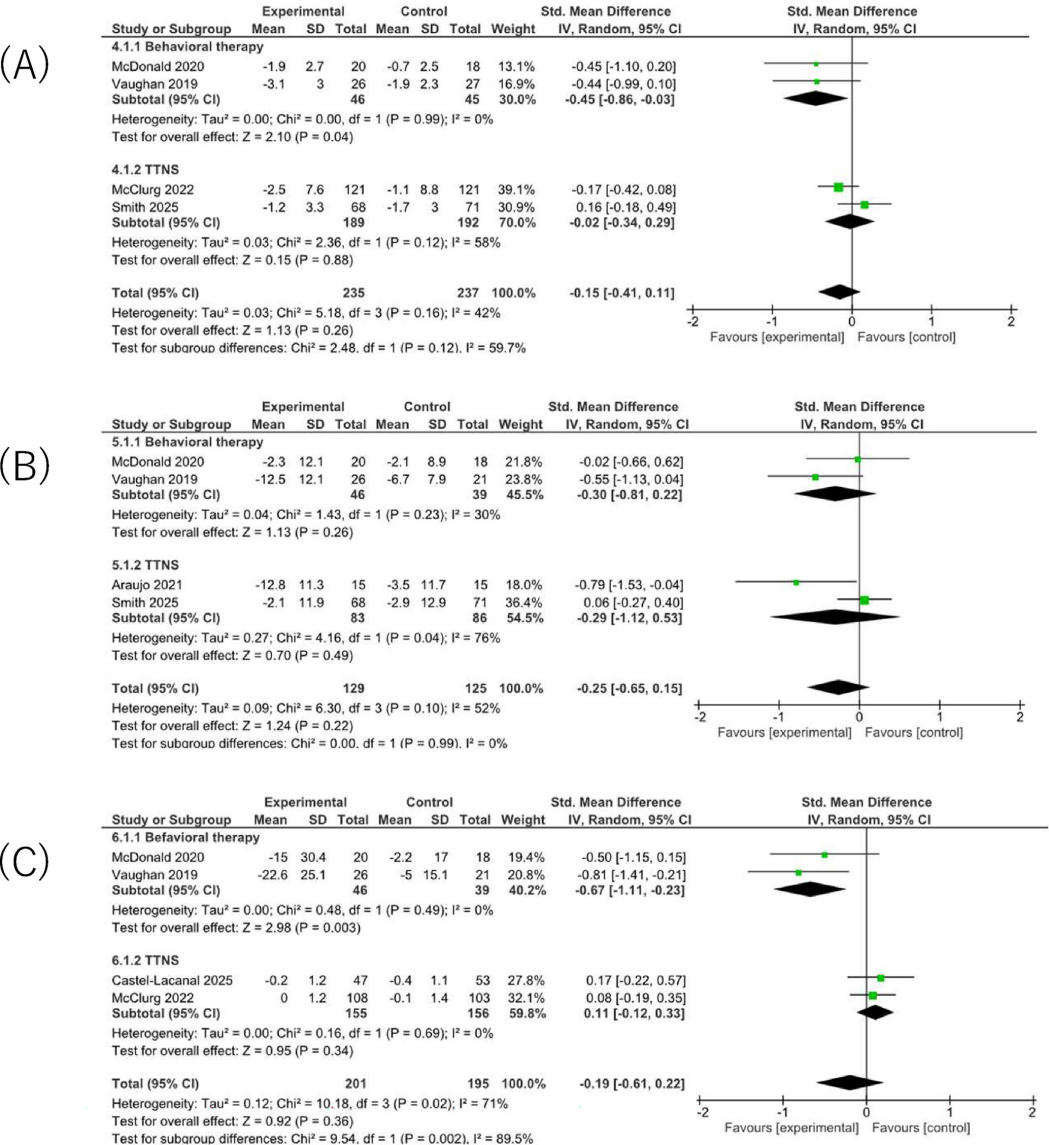
Forest plots analysis of questionnaire-based outcomes. (A) Symptom scores (International Consultation on Incontinence Questionnaire–Overactive Bladder [ICIQ-OAB] symptom score, International Prostate Symptom Score [IPSS]). (B) Bother scores (ICIQ-OAB bother score, Overactive Bladder Questionnaire short form [OAB-V8]). (C) Urinary-related quality-of-life scores (ICIQ-OAB QoL, SF-Qualiveen overall score).

For urinary symptom scores, the pooled estimate suggested a small improvement with NPNS interventions, but the CI included no important difference (SMD −0.15, 95% CI −0.41 to 0.11; Fig. 4A) [33], [35], [39], [41]. Heterogeneity was moderate (τ^2^ = 0.03, I^2^ = 42%). The certainty of evidence was low.

#### 3.6.2 Urinary bother scores

For urinary bother scores, the pooled estimate favored NPNS interventions but was imprecise (SMD −0.25, 95% CI −0.65 to 0.15; Fig. 4B) [33], [35], [37], [41]. Heterogeneity was moderate (τ^2^ = 0.09, I^2^ = 52%). The certainty of evidence was low.

#### 3.6.3 Urinary-related QOL

For urinary-related QOL, the pooled estimate favored NPNS interventions but was imprecise, with the 95% CI spanning both benefit and no important difference (SMD −0.19, 95% CI −0.61 to 0.22; Fig. 4C) [33], [35], [38], [39]. Between-study heterogeneity was substantial (τ^2^ = 0.00, I^2^ = 71%), and this pooled estimate is therefore presented as an exploratory summary. The certainty of evidence was very low.

#### 3.6.4 Movement Disorder Society-Unified Parkinson’s Disease Rating Scale’ urinary problem

For the Movement Disorder Society-Unified Parkinson’s Disease Rating Scale (MDS-UPDRS) urinary item, the pooled estimate favored NPNS interventions (MD −0.50, 95% CI −0.82 to −0.17; Supplementary Appendix S10) [29], [30], [31], [43]. Heterogeneity was low (τ^2^ = 0.02, I^2^ = 18%). The certainty of evidence was moderate.

#### 3.6.5 Non-motor symptoms scale domain 7: urinary

One study evaluating caloric vestibular stimulation estimated an improvement in the non-motor symptoms scale (NMSS) urinary domain (MD −5.30, 95% CI −10.09 to −0.51; Supplementary Appendix S10) [43]. The certainty of evidence for this outcome was very low.

### 3.7 Sensitivity analysis

Primary analyses were based on postintervention values. A sensitivity analysis was conducted for voids per 24 hr because one study reported a derived outcome rather than directly reported daily void counts. Sensitivity analyses using imputed change-score SDs (assumed correlation coefficients r = 0, 0.5, and 1) yielded conclusions consistent with the primary analysis (Supplementary Appendix S9). Sensitivity analyses were not feasible for other outcomes because postintervention data were not available or because the number of contributing studies was insufficient.

### 3.8 Reporting bias

Across outcomes, the number of studies contributing to each meta-analysis was too small to support informative assessment of reporting bias. Therefore, funnel plots were not used, and potential concerns were considered qualitatively as possible small-study effects when interpreting certainty of evidence.

### 3.9 Certainty of evidence

The certainty of evidence for each pooled outcome was assessed using GRADE (Table 1). Certainty was rated as moderate for incontinence episodes; low for urgency episodes, urinary symptom scores, and bother scores; and very low for number of voids and urinary-related QOL. For supplementary urinary-related outcomes, certainty was moderate for the MDS-UPDRS urinary item and very low for the NMSS urinary domain.

**Table 1:** Summary of findings

	Certainty assessment							№ of patients		Effect Absolute(95% CI)	Certainty
Outcome	№ of studies	Study design	Risk of bias	Inconsistency	Indirectness	Imprecision	Other considerations	Non-pharmacological and Non-surgical intervention	Control		
Number of voids	2	randomised trials	not serious	very serious^a^	not serious	serious^b^	none	132	130	MD **0.68 SD lower** (2.43 lower to 1.07 higher)	⊕○○○ Very low^a^, ^b^
Urgency episodes	3	randomised trials	not serious	serious^c^	not serious	serious^b^	none	149	143	MD **1.42 SD lower** (3 lower to 0.16 higher)	⊕⊕○○ Low^b^, ^c^
Incontinence episodes	4	randomised trials	not serious	serious^c^	not serious	not serious	none	175	164	MD **0.91 SD lower** (1.74 lower to 0.08 lower)	⊕⊕⊕○ Moderate^c^
Symptom score	4	randomised trials	not serious	serious^c^	not serious^c^	serious^b^	none	235	237	SMD **0.15 SD lower** (0.41 lower to 0.11 higher)	⊕⊕○○ Low^b^, ^c^
Bother score	4	randomised trials	not serious	serious^c^	not serious	serious^b^	none	129	125	SMD **0.25 SD lower** (0.65 lower to 0.15 higher)	⊕⊕○○ Low^b^, ^c^
QOL score	4	randomised trials	not serious	very serious^d^	not serious^e^	serious^b^	none	201	195	SMD **0.19 SD lower** (0.61 lower to 0.22 higher)	⊕○○○ Very low^b^, ^d^, ^e^
MDS-UPDRS	4	randomised trials	not serious	not serious	serious^f^	not serious	none	71	71	MD **0.5 lower** (0.82 lower to 0.17 lower)	⊕⊕⊕○ Moderate^f^
Non-Motor Symptom Scale: Domain 7 urinary	1	randomised trials	not serious	not serious	serious^f^	very serious^g^, ^h^	none	16	17	MD **5.3 lower** (10.09 lower to 0.51 lower)	⊕○○○ Very low^f^, ^g^, ^h^

CI = confidence interval; MD = mean difference; SMD = standardized mean difference.

a Moderate heterogeneity.

b Confidence interval is wide and includes opposite effects.

c Downgraded one level for inconsistency due to heterogeneity by intervention subtype.

d Severe statistical heterogeneity.

e Downgraded two levels for very serious inconsistency due to extreme heterogeneity and conflicting directions of effect across studies.

f Uncertain presence of LUTS in Parkinson’s disease limits evidence applicability.

g Total sample size is small.

h Confidence intervals is wide.

## 4 Discussion

### 4.1 Principal findings

This systematic review and meta-analysis examined the effects of NPNS interventions on LUTS in people with PD. Overall, the findings suggest that these interventions may improve certain urinary outcomes; however, effect estimates were generally imprecise and characterized by substantial between-study heterogeneity, resulting in low to very low certainty of evidence for most outcomes. Among the evaluated outcomes, urinary incontinence episodes showed the most consistent signal, with a modest reduction supported by moderate certainty of evidence. In contrast, urgency episodes, symptom scores, and bother scores demonstrated possible but uncertain benefits, while effects on voiding frequency and urinary-related QOL remained highly uncertain.

### 4.2 Interpretation in the context of heterogeneity and certainty

A key feature of the evidence base was the considerable heterogeneity observed across several pooled outcomes. Heterogeneity was assessed using both I^2^ and τ^2^, allowing evaluation of relative inconsistency and absolute between-study variability on the effect-size scale. The presence of substantial heterogeneity indicates that pooled estimates should be interpreted as conditional summaries rather than precise estimates of a uniform treatment effect. In many outcomes, wide CIs spanning both benefit and no important difference further limited interpretability and contributed to downgrading of certainty for imprecision.

The comparatively higher certainty observed for urinary incontinence episodes suggests that this outcome may be more sensitive to NPNS interventions than other LUTS domains. In contrast, outcomes such as voiding frequency and QOL may be influenced by greater variability in outcome definitions, measurement instruments, and study context, which likely diluted pooled estimates and increased uncertainty. These findings underscore the importance of aligning outcome selection and measurement strategies with clinically meaningful and intervention-responsive domains.

### 4.3 Heterogeneity and the role of exploratory subgroup analyses

The substantial heterogeneity observed across multiple outcomes raises the possibility that treatment effects may be context-dependent rather than uniform across studies. Differences in intervention characteristics, comparator conditions, and assessment timing may contribute to variability in observed effects. To explore these possibilities, subgroup analyses were conducted and are reported in the Supplementary Appendix. However, these analyses were exploratory and underpowered, and therefore cannot be used to establish differential efficacy.

Rather than providing definitive explanations, the subgroup findings highlight potential sources of effect modification that warrant prospective evaluation. Future studies designed a priori to test interaction effects, with adequate sample sizes and standardized outcome measures, are needed to determine whether specific intervention characteristics or clinical contexts modify treatment response.

### 4.4 Comparison with existing literature

The overall pattern of findings is broadly consistent with previous literature suggesting potential benefits of conservative and rehabilitative approaches for urinary symptoms in PD, while also highlighting persistent uncertainty [2], [22]. Prior studies and reviews have reported mixed effects across LUTS domains, often limited by small sample sizes and heterogeneous methodologies [2], [33], [35]. The present review extends this literature by systematically evaluating heterogeneity using both relative and absolute metrics and by integrating certainty-of-evidence assessments to contextualize pooled estimates. The findings reinforce the view that NPNS interventions may offer symptom relief for some patients, but that current evidence remains insufficient to support broad or definitive conclusions across all urinary outcomes.

### 4.5 Clinical and research implications

From a clinical perspective, the results suggest that NPNS interventions may be considered as part of a comprehensive management strategy for urinary symptoms in PD, particularly for urinary incontinence. However, given the low to very low certainty for most outcomes, these interventions should be applied with appropriate caution, and expectations regarding magnitude and consistency of benefit should remain modest.

For research, the findings emphasize the need for well-designed trials with clearer specification of intervention components, standardized outcome definitions, and consistent assessment timing. Greater emphasis on clinically meaningful endpoints and transparent reporting of comparator conditions may reduce heterogeneity and improve interpretability. Importantly, future studies should be designed to test potential effect modifiers explicitly, rather than relying on post hoc exploratory subgroup analyses.

### 4.6 Strengths and limitations

This review has several strengths, including a comprehensive search strategy, transparent data extraction procedures, and rigorous assessment of heterogeneity and certainty of evidence using established frameworks. The use of both I^2^ and τ^2^ facilitated a more nuanced interpretation of between-study variability, and detailed analyses were made available in the Supplementary Appendix to support transparency.

Nevertheless, the findings should be interpreted in light of important limitations. The number of studies contributing to each outcome was often small, limiting statistical power and the ability to explore heterogeneity conclusively. Variability in intervention content, outcome measurement, and study design contributed to imprecision and indirectness, which reduced certainty for several outcomes. These limitations reflect the current state of the evidence base and highlight priorities for future research.

These limitations may have influenced both the apparent direction and magnitude of pooled effects, particularly for subjective outcomes where incomplete blinding may increase performance/detection bias and for outcomes with heterogeneous comparators and measurement approaches that contribute to inconsistency and imprecision.

Accordingly, pooled estimates should be interpreted cautiously as possible benefit rather than confirmed efficacy.

## 5 Conclusions

This systematic review suggests that NPNS interventions may improve certain LUTS in people with PD; however, the certainty of evidence varies substantially across outcomes. Among the evaluated domains, urinary incontinence episodes showed the most consistent signal, supported by moderate-certainty evidence. In contrast, urgency episodes, urinary symptom scores, and bother scores indicated possible benefit with low certainty, while effects on voiding frequency, urinary-related QOL, and the NMSS urinary domain remained highly uncertain. Effect estimates for many outcomes were imprecise and characterized by substantial between-study heterogeneity, assessed using both τ^2^ and I^2^, indicating that pooled estimates should be interpreted as conditional summaries rather than definitive effects. Exploratory subgroup and sensitivity analyses did not materially alter these conclusions. Overall, current evidence supports cautious interpretation of potential benefits, with confirmation requiring further well-designed studies.

  ***Author contributions:*** Hiroyuki Ohtsuka had full access to all the data in the study and takes responsibility for the integrity of the data and the accuracy of the data analysis.

  *Study concept and design:* Ohtsuka, Ouchi, Otsuka, Kitta, Sakakibara.

*Acquisition of data:* Ohtsuka, Ouchi, Otsuka, Kitta, Sakakibara.

*Analysis and interpretation of data:* Ohtsuka, Ouchi, Otsuka, Kitta, Yoneoka, Sakakibara.

*Drafting of the manuscript:* Ohtsuka, Ouchi.

*Critical revision of the manuscript for important intellectual content:* Kitta, Yoneoka, Sakakibara.

*Statistical analysis:* Ohtsuka, Yoneoka.

*Obtaining funding:* Ohtsuka.

*Administrative, technical, or material support:* None.

*Supervision:* Kitta, Sakakibara.

*Other:* None.

  ***Funding/Support and role of the sponsor:*** The authors acknowledge the internal funding provided by Showa University for supporting this study.

  ***Financial disclosures:*** Hiroyuki Ohtsuka certifies that all conflicts of interest, including specific financial interests and relationships and affiliations relevant to the subject matter or materials discussed in the manuscript (eg, employment/affiliation, grants or funding, consultancies, honoraria, stock ownership or options, expert testimony, royalties, or patents filed, received, or pending), are the following: The authors declare that they have no conflict of interest.

  ***Acknowledgment:*** We would like to thank Editage (www.editage.com) for providing English language editing support during the preparation of this manuscript.

  ***Data availability statement:*** The data supporting the findings of this study are available from the corresponding author upon reasonable request. Extracted study-level data used for meta-analyses (effect estimates, SD conversions, and outcome-direction coding) and the outcome dictionary are provided in the Supplementary Appendices. Additional analytic files are available from the corresponding author upon reasonable request.

  ***Declaration of Generative AI and AI-assisted technologies in the writing process:*** During the preparation of this work the author(s) used ChatGPT (OpenAI) to improve the clarity and structure of the English language. After using this tool/service, the author(s) reviewed and edited the content as needed and take(s) full responsibility for the content of the publication.
