# Supplementary material for: Effects of Nonpharmacological and Nonsurgical Intervention for Lower Urinary Tract Symptoms in Parkinson’s Disease: A Systematic Review and Meta-analysis
Source: Eur Urol Open Sci. 2026 Apr 9;87:80–90. doi: 10.1016/j.euros.2026.03.009 (PMC13091170; doi:10.1016/j.euros.2026.03.009)
Supplement: Supplementary Appendix S1–S10 [file mmc1.docx]

**Supplementary Appendix S1**

**Search Update History**

Supplementary Appendix S1 provides the history of the literature search updates conducted during the review process.

The initial search was performed in September 2021 and was subsequently updated in September 2024 and November 2025 to ensure currency. The eligibility criteria, databases searched, and core search logic remained unchanged across updates. The final report reflects the search conducted on 28 November 2025.

**Supplementary Appendix S2**

**Full Search Strategy**

Supplementary Appendix S2 presents the full electronic search strategy used for each database.

This appendix details the complete search strings, including all key terms related to the target population, interventions, and outcomes, as well as the Boolean logic (AND/OR) applied. The information provided enables full reproducibility of the search process.

(1) PubMed

| Search number | Query |
| --- | --- |
| 1 | ("Lewy Body Disease"[Mesh] OR "Lewy"[All Fields]) |
| 2 | ("Parkinson Disease"[Mesh] OR ("Parkinson" AND "disease")[All Fields] OR "parkinson disease"[All Fields] OR "Parkinson's disease"[All Fields] OR "Parkinsonian"[All Fields]) |
| 3 | #1 OR #2 |
| 4 | (("Lower Urinary Tract Symptoms"[Mesh]) OR ("nocturia"[All Fields] ) OR ("Urinary"[All Fields] ) OR ("Bladder"[All Fields] ) OR ("overactive"[All Fields] ) OR ("incontinence"[All Fields] ) OR ("Urine"[All Fields] ) OR (Urin*[All Fields] ) OR (Urge*[All Fields] ) OR ("Stress"[All Fields] ) OR ("non-motor"[All Fields]) OR ("nonmotor"[All Fields])) |
| 5 | #3 AND #4 |
| 6 | (("Diet, Food, and Nutrition"[Mesh]) OR ("Beverages"[Mesh]) OR ("Life Style"[Mesh]) OR ("Weight Reduction Programs"[Mesh]) OR ("Caffeine"[Mesh]) OR ("Smoking"[Mesh]) OR ("physical activity"[All Fields]) OR (physical*[All Fields]) OR (activ*[All Fields]) OR ("diet"[All Fields])) |
| 7 | (("Exercise"[Mesh]) OR ("Pelvic Floor"[Mesh]) OR ("Exercise Therapy"[Mesh]) OR ("Rehabilitation"[Mesh]) OR ("Physical Therapy Modalities "[Mesh]) OR ("pelvic"[All Fields]) OR ("rehabilitation"[All Fields]) OR ("physical therapy"[All Fields]) OR ("physiotherapy"[All Fields]) OR ("training"[All Fields]) OR ("feedback"[All Fields]) OR ("biofeedback"[All Fields])) |
| 8 | (("Behavior Therapy"[Mesh]) OR ("Behavior and Behavior Mechanisms"[Mesh]) OR ("voiding"[All Fields]) OR ("bladder"[All Fields]) OR (schedule [All Fields]) OR (time[All Fields]) OR (habit [All Fields]) OR (prompt [All Fields])) |
| 9 | (("Electric Stimulation Therapy"[Mesh]) OR ("Electric Stimulation"[Mesh]) OR ("transcutaneous"[All Fields]) OR (electric* [All Fields])　OR ("tibial"[All Fields]) OR ("peripherall"[All Fields])) |
| 10 | (("Transcranial Direct Current Stimulation"[Mesh]) OR ("Transcranial Magnetic Stimulation"[Mesh]) OR ("tDCS"[All Fields]) OR ("TMS"[All Fields]) OR ("rTMS"[All Fields]) OR ("transcranial"[All Fields]) OR ("magnetic"[All Fields])) |
| 11 | #6 OR #7 OR #8 OR #9 OR #10 |
| 12 | #5 AND #11 |
| 13 | #12 NOT (Animals [Mesh] NOT Humans[Mesh]) |
| 14 | #14 AND "Study Characteristics" [Publication Type] |

(2) Cochrane Central Register of Controlled Trials

| Search number | Query |
| --- | --- |
| 1 | MeSH descriptor: [Lewy Body Disease] explode all trees |
| 2 | Lewy |
| 3 | MeSH descriptor: [Parkinson Disease] explode all trees |
| 4 | ("Parkinson" AND "disease") OR "parkinson disease" OR "Parkinson's disease" OR "Parkinsonian" |
| 5 | MeSH descriptor: [Lower Urinary Tract Symptoms] explode all trees |
| 6 | nocturia OR "Urinary" OR "Bladder" OR "overactive" OR "incontinence" OR "Urine" OR Urin* OR Urge* OR "Stress" OR "non-motor" OR "nonmotor" |
| 7 | MeSH descriptor: [Diet, Food, and Nutrition] explode all trees |
| 8 | MeSH descriptor: [Beverages] explode all trees |
| 9 | MeSH descriptor: [Life Style] explode all trees |
| 10 | MeSH descriptor: [Weight Reduction Programs] explode all trees |
| 11 | MeSH descriptor: [Caffeine] explode all trees |
| 12 | MeSH descriptor: [Smoke] explode all trees |
| 13 | physical activity OR physical* OR activ* OR "diet" |
| 14 | MeSH descriptor: [Exercise] explode all trees |
| 15 | MeSH descriptor: [Pelvic Floor] explode all trees |
| 16 | MeSH descriptor: [Exercise Therapy] explode all trees |
| 17 | MeSH descriptor: [Rehabilitation] explode all trees |
| 18 | MeSH descriptor: [Physical Therapy Modalities] explode all trees |
| 19 | pelvic OR "rehabilitation" OR "physical therapy" OR "physiotherapy" OR "training" OR "feedback" OR "biofeedback" |
| 20 | MeSH descriptor: [Behavior Therapy] explode all trees |
| 21 | MeSH descriptor: [Behavior and Behavior Mechanisms] explode all trees |
| 22 | voiding OR "bladder" OR schedule OR time OR habit OR prompt |
| 23 | MeSH descriptor: [Electric Stimulation Therapy] explode all trees |
| 24 | MeSH descriptor: [Electric Stimulation] explode all trees |
| 25 | transcutaneous OR electric* OR "tibial" OR "peripherall" |
| 26 | MeSH descriptor: [Transcranial Direct Current Stimulation] explode all trees |
| 27 | MeSH descriptor: [Transcranial Magnetic Stimulation] explode all trees |
| 28 | tDCS OR "TMS" OR "rTMS" OR "transcranial" OR "magnetic" |
| 29 | (#1 OR #2 OR #3 OR #4) |
| 30 | (#5 OR #6) |
| 31 | (#7 OR #8 OR #9 OR #10 OR #11 OR #12 OR #13 OR #14 OR #15 OR #16 OR #17 OR #18 OR #19 OR #20 OR #21 OR #22 OR #23 OR #24 OR #25 OR #26 OR #27 OR #28) |
| 32 | #29 AND #30 AND #31 |

(3) CINAHL

| Search number | Query |
| --- | --- |
| 1 | (MH "Lewy Body Disease") OR "Lewy" |
| 2 | (MH "Parkinson Disease") OR ("Parkinson" AND "disease") OR "parkinson disease" OR "Parkinson's disease" OR "Parkinsonian") |
| 3 | #1 OR #2 |
| 4 | (MH "Urinary Elimination Alteration (Saba CCC)") OR ("nocturia") OR ("Urinary") OR ("Bladder") OR ("overactive") OR ("incontinence") OR ("Urine") OR (Urin*) OR (Urge*) OR ("Stress") OR ("non-motor") OR ("nonmotor") |
| 5 | #3 AND #4 |
| 6 | (MH "Diet") OR (MH "Nutrition") OR (MH "Food") OR (MH "Beverages") OR (MH "Life Style") OR (MH "Weight Reduction Programs") OR (MH "Caffeine") OR (MH "Smoking") OR ("physical activity") OR (physical*) OR (activ*) OR ("diet") |
| 7 | (MH "Exercise") OR (MH "Pelvic Floor Muscles") OR (MH "Rehabilitation") OR (MH "Physical Therapy") OR ("pelvic") OR ("rehabilitation") OR ("physical therapy") OR ("physiotherapy") OR ("training") OR ("feedback") OR ("biofeedback") |
| 8 | (MH "Behavior Therapy") OR (MH "Behavior Therapy (Iowa NIC)") OR (MH "Behavior") OR ("Behavior and Behavior Mechanisms"[Mesh]) OR ("voiding") OR ("bladder") OR (schedule) OR (time) OR (habit) OR (prompt) |
| 9 | (MM "Electrical Stimulation, Functional") OR (MM "Electrical Stimulation, Neuromuscular") OR (MM "Transcutaneous Electrical Nerve Stimulation (Iowa NIC)") OR (MM "Electric Stimulation+") OR (MM "Transcutaneous Electric Nerve Stimulation") OR ("transcutaneous") OR (electric*)　OR ("tibial") OR ("peripherall") OR (“stimulation”) |
| 10 | (MH "Transcranial Magnetic Stimulation") OR (MH "Transcranial Direct Current Stimulation") OR ("tDCS") OR ("TMS") OR ("rTMS") OR ("transcranial") OR ("magnetic") |
| 11 | #6 OR #7 OR #8 OR #9 OR #10 |
| 12 | #5 AND #11 |

(4) CINAHL

| Search number | Query |
| --- | --- |
| 1 | Lewy |
| 2 | Parkinson* |
| 3 | Diet |
| 4 | Food |
| 5 | Nutrition |
| 6 | Beverages |
| 7 | Life Style |
| 8 | Weight Reduction Programs |
| 9 | Caffeine |
| 10 | Smoke |
| 11 | physical |
| 12 | Exercise |
| 13 | Pelvic |
| 14 | Voiding |
| 15 | Bladder |
| 16 | Stimulation |
| 17 | #1 AND #3 |
| 18 | #1 AND #4 |
| 19 | #1 AND #5 |
| 20 | #1 AND #6 |
| 21 | #1 AND #7 |
| 22 | #1 AND #8 |
| 23 | #1 AND #9 |
| 24 | #1 AND #10 |
| 25 | #1 AND #11 |
| 26 | #1 AND #12 |
| 27 | #1 AND #13 |
| 28 | #1 AND #14 |
| 29 | #1 AND #15 |
| 30 | #1 AND #16 |
| 31 | #2 AND #3 |
| 32 | #2 AND #4 |
| 33 | #2 AND #5 |
| 34 | #2 AND #6 |
| 35 | #2 AND #7 |
| 36 | #2 AND #8 |
| 37 | #2 AND #9 |
| 38 | #2 AND #10 |
| 39 | #2 AND #11 |
| 40 | #2 AND #12 |
| 41 | #2 AND #13 |
| 42 | #2 AND #14 |
| 43 | #2 AND #15 |
| 44 | #2 AND #16 |
| 45 | #17 OR #18 OR #19 OR #20 OR #21 OR #22 OR #23 OR #24 OR #25 OR #26 OR #27 OR #28 OR #29 OR #30 OR #31 OR #32 OR #33 OR #34 OR #35 OR #36 OR #37 OR #38 OR #39 OR #40 OR #41 OR #42 OR #43 OR #44 |

**Supplementary Appendix S3**

**Excluded Full-text Articles with Reasons**

Supplementary Appendix S3 lists all full-text articles assessed for eligibility but excluded from the final review.

For each excluded study, the primary reason for exclusion is reported to ensure transparency in the study selection process.

This appendix is provided as a separate Excel file to facilitate readability and inspection of exclusion decisions.

**Supplementary Appendix S4**

**Outcome Dictionary and Instrument Specifications**

Supplementary Appendix S4 provides a detailed outcome dictionary and instrument-level specifications.

When multiple measures or time points were reported for the same outcome domain within a study, a single effect estimate was selected according to a predefined outcome hierarchy and end-of-treatment prioritisation, to avoid unit-of-analysis errors.

This appendix describes all outcome domains considered in the review, the instruments used to measure each domain, the orientation of each scale, the direction-of-benefit coding, and the predefined hierarchy applied to select a single outcome per domain for quantitative synthesis.

This appendix is provided as a separate Excel file to facilitate clear presentation and systematic inspection of outcome definitions and coding rules.

**Supplementary Appendix S5**

**Protocol Deviations from PROSPERO Registration**

Supplementary Appendix S5 documents all deviations from the prespecified PROSPERO protocol.

For each deviation, the original plan, the modification implemented during the review process, the rationale for the change, and its implications for interpretation are reported in accordance with PRISMA item 24c.

This appendix is provided as a separate Excel file to allow structured presentation and transparent review of protocol deviations.

**Supplementary Appendix S6**

**Additional Statistical Methods and Sensitivity Analyses**

Supplementary Appendix S6 describes additional statistical methods and sensitivity analyses not fully detailed in the main Methods section.

This appendix includes the rationale and procedures for imputation of missing standard deviations, handling of derived bladder-diary outcomes, and sensitivity analyses conducted to assess the robustness of pooled estimates.

**1. Imputation of Missing Standard Deviations for Change Scores**

In several studies, standard deviations (SDs) for change scores were not reported. When change scores were required for sensitivity analyses and corresponding SDs were unavailable, we imputed SDs using the method recommended in the Cochrane Handbook for Systematic Reviews of Interventions. Specifically, SDs for change scores were calculated assuming plausible correlations (r) between baseline and post-intervention values, using the following formula:

$$SD_{\text{change}}=\sqrt{SD_{\text{baseline}}^{2}+SD_{\text{post}}^{2}-2r\times SD_{\text{baseline}}\times SD_{\text{post}}}$$

To assess the robustness of results to this assumption, sensitivity analyses were conducted using three prespecified correlation values (r = 0, 0.5, and 1). These analyses were performed only when post-intervention SDs were not available and when at least two studies contributed data to the pooled estimate.

**2. Handling of Derived Bladder-Diary Outcomes**

Some studies did not directly report bladder-diary outcomes as totals per 24 hours but instead provided separate values for daytime and nocturnal voiding episodes. In such cases, total daily voids were derived by summing daytime and nocturnal voids. Because these values were not directly reported as a single 24-hour outcome in the original studies, they were considered derived outcomes.

Derived bladder-diary outcomes were retained in the primary meta-analyses when the underlying construct was judged to be equivalent across studies. However, to account for potential heterogeneity arising from differences in outcome construction, these derived outcomes were explicitly flagged and examined in sensitivity analyses.

**3. Sensitivity Analysis Procedures**

Sensitivity analyses were prespecified to evaluate the robustness of pooled estimates to key analytical decisions, including (i) the inclusion of derived bladder-diary outcomes and (ii) the imputation of SDs for change scores. Sensitivity analyses were conducted only when methodologically applicable and feasible, defined a priori as the availability of at least two studies contributing to the analysis.

When sensitivity analyses were not feasible (e.g., because sufficient post-intervention data were available or because too few studies were included), this was explicitly stated in the Results section, and no additional analyses were performed.

**4. Interpretation of Sensitivity Analyses**

The results of sensitivity analyses were interpreted qualitatively, focusing on the consistency of effect direction and the magnitude and overlap of 95% confidence intervals, rather than on changes in pooled point estimates alone. Sensitivity analyses were used to assess whether key analytical assumptions materially influenced the overall conclusions of the review.

**5. Rationale for Analytical Choices for Count Outcomes**

Bladder-diary outcomes represent count data and are, in principle, amenable to Poisson or negative binomial modeling. However, because individual participant data were not available and original studies did not consistently report rate-based effect measures, reanalysis using count regression models was not feasible. Accordingly, these outcomes were analyzed using a continuous approximation, and pooled estimates were interpreted cautiously as summary measures rather than rate ratios.

**Supplementary Appendix S8**

**Study Characteristics**

Supplementary Appendix S8 provides detailed study characteristics for all included studies.

This appendix summarizes study design, sample size, participant characteristics, intervention and comparator details, outcome definitions and assessment timing, and adverse event reporting.

This appendix is provided as a separate Excel file to facilitate clear presentation and systematic inspection of study-level characteristics that exceed feasible length for inclusion in a Word-based appendix.

**Supplementary Appendix S9**

**Sensitivity Analyses and Exploratory Subgroup Analyses**

Supplementary Appendix S9 reports sensitivity analyses and exploratory subgroup analyses conducted to support interpretation of between-study heterogeneity.

**S9.1 Sensitivity Analyses**

**S9.1.1 Rationale for Sensitivity Analyses**

Sensitivity analyses were conducted to assess the robustness of pooled estimates where outcome construction or data format differed across studies.

**S9.1.2 Sensitivity Analysis for Number of Voids per 24 Hours**

Forest plots of sensitivity analyses for the number of voids using imputed change-score standard deviations. (A) r = 0, (B) r = 0.5, and (C) r = 1. Correlation coefficients were varied to represent a plausible range of baseline–post correlations. Sensitivity analyses were conducted because Araujo et al. (2021) reported the number of voids as a derived outcome based on the sum of daytime and nocturnal voids.


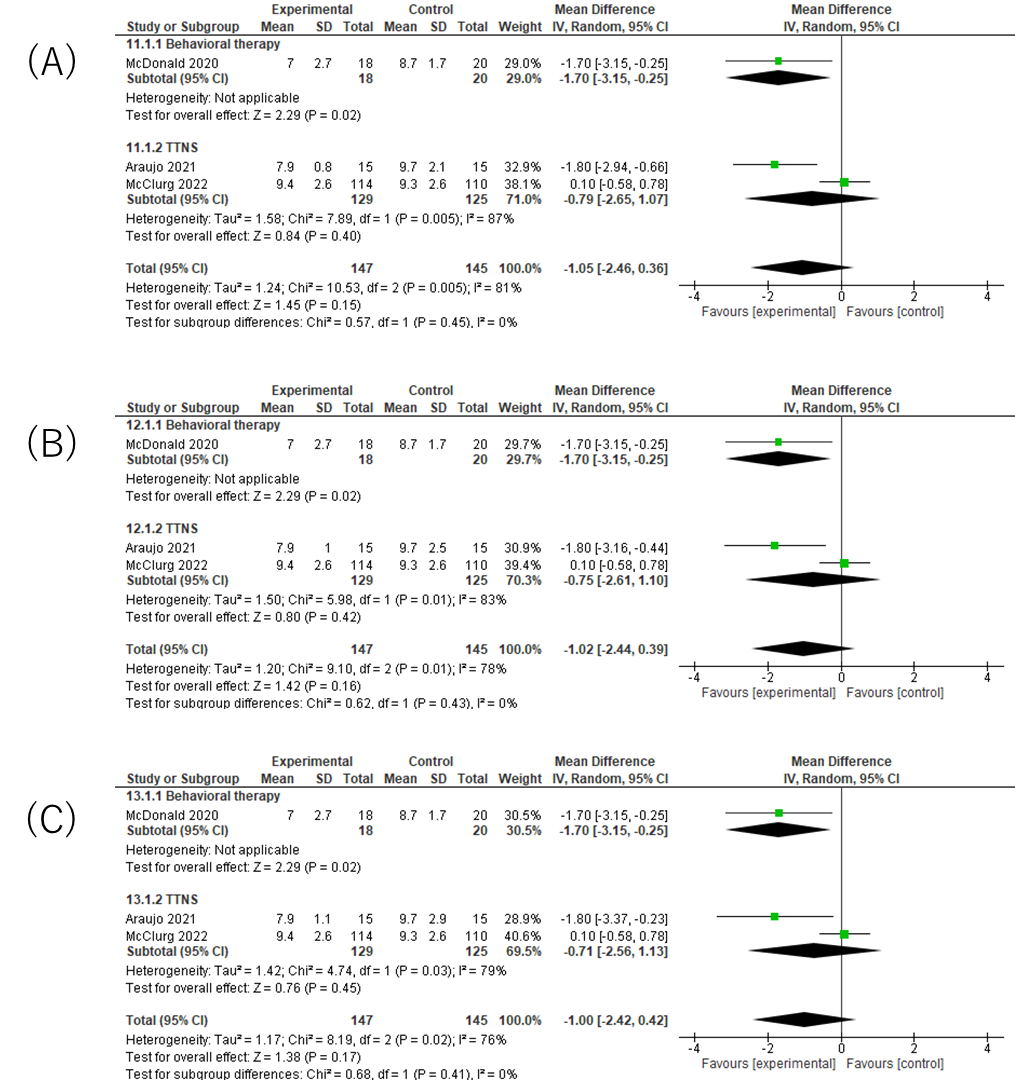


**S9.1.3 Sensitivity Analyses for Other Outcomes**

Sensitivity analyses were not feasible for other outcomes because post-intervention mean and standard deviation values were available for pooling and/or the number of contributing studies per outcome was insufficient to support additional robustness checks.

**S9.2 Exploratory Subgroup Analyses**

**S9.2.1 General Approach to Subgroup Analyses**

Exploratory subgroup analyses were conducted to contextualize between-study heterogeneity.
All subgroup analyses were underpowered, hypothesis-generating, and not intended to establish differential efficacy.

**S9.2.2 Subgroup Analyses by Intervention Type**

Subgroup analyses by intervention type were conducted to explore whether between-study heterogeneity could be contextualized according to broad intervention categories.

Intervention types were grouped as behavioral therapy–based interventions and transcutaneous tibial nerve stimulation (TTNS). All subgroup analyses were exploratory and underpowered and are presented to aid interpretation of heterogeneity rather than to establish differential efficacy.

**Number of voids per 24 hours (Figure 3A)**

In subgroup analyses, behavioral therapy–based interventions showed a larger estimated reduction in voids per 24 hours (MD –1.70, 95% CI –3.15 to –0.25), whereas the TTNS subgroup estimate was imprecise and compatible with both benefit and no important difference (MD 0.10, 95% CI –0.58 to 10.78).
These subgroup estimates were derived from a small number of trials using different comparators and outcome constructions and should therefore be interpreted cautiously.

**Episodes of urgency (Figure 3B)**

For urgency episodes per 24 hours, subgroup estimates were imprecise for both intervention categories.
The behavioral therapy subgroup yielded an estimated MD of –0.90 (95% CI –2.66 to 0.86), while the TTNS subgroup yielded an estimated MD of –1.63 (95% CI –3.88 to 0.63).
Substantial overlap of confidence intervals and limited numbers of contributing studies preclude meaningful inference regarding differential effects between intervention types.

**Episodes of incontinence (Figure 3C)**

In subgroup analyses of urinary incontinence episodes per 24 hours, behavioral therapy–based interventions showed a small estimated reduction (MD –0.74, 95% CI –1.41 to –0.06).
The TTNS subgroup estimate was more uncertain (MD –1.13, 95% CI –2.80 to 0.53), reflecting wider confidence intervals and greater between-study variability.
These findings should be interpreted as exploratory signals only.

**Urinary symptom scores (Figure 4A)**

For urinary symptom scores, subgroup analyses suggested a larger estimated improvement with behavioral therapy–based interventions (SMD –0.45, 95% CI –0.86 to –0.03), whereas the TTNS subgroup estimate was close to no difference and imprecise (SMD –0.02, 95% CI –0.34 to 0.29).
Given the subjective nature of symptom scores and incomplete blinding in some trials, these subgroup estimates should be interpreted cautiously.

**Urinary bother scores (Figure 4B)**

For urinary bother scores, subgroup estimates were imprecise for both intervention categories.
The behavioral therapy subgroup yielded an estimated SMD of –0.30 (95% CI –0.81 to 0.22), and the TTNS subgroup yielded an estimated SMD of –0.29 (95% CI –1.12 to 0.53).
Overlapping confidence intervals and limited sample sizes limit interpretation of subgroup differences.

**Urinary-related quality of life (Figure 4C)**

In subgroup analyses of urinary-related quality of life, behavioral therapy–based interventions showed a larger estimated improvement (SMD –0.67, 95% CI –1.11 to –0.23), whereas the TTNS subgroup estimate was close to no difference and uncertain (SMD 0.11, 95% CI –0.12 to 0.33).
Considerable heterogeneity and variation in QOL instruments across studies further limit interpretation of these subgroup findings.

**Summary (Subgroup by Intervention Type)**

Across outcomes, exploratory subgroup analyses by intervention type suggested that between-study heterogeneity may be influenced by differences in intervention content and delivery.
However, given the small number of studies within each subgroup, heterogeneity in comparators and outcome definitions, and overlapping confidence intervals, these analyses should be interpreted cautiously and are not intended to support conclusions regarding differential efficacy.

**Supplementary Appendix S10**

Forest plot analysis of the outcome of the urinary part of the (A) MDS-UPDRS and (B)NMSS


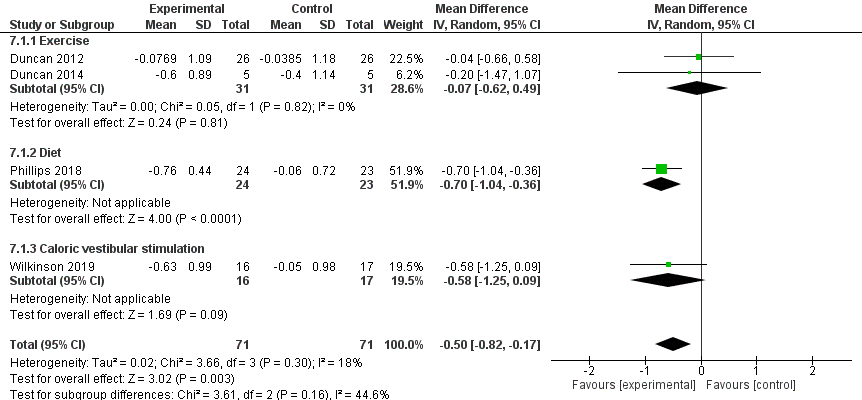
(A)


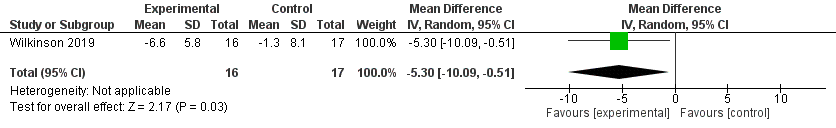
(B)
